# The Flow of Glasses and Glass–Liquid Transition under Electron Irradiation

**DOI:** 10.3390/ijms241512120

**Published:** 2023-07-28

**Authors:** Michael I. Ojovan

**Affiliations:** 1 Department of Materials, Imperial College London, London SW7 2AZ, UK; m.ojovan@imperial.ac.uk; 2 Department of Materials Science and Engineering, University of Sheffield, Sheffield S1 3JD, UK

**Keywords:** glass, glass transition, irradiation, dose rate, configuron, percolation, activation energy

## Abstract

Recent discovery and investigation of the flow of glasses under the electron beams of transmission electron microscopes raised the question of eventual occurrence of such type effects in the vitrified highly radioactive nuclear waste (HLW). In connection to this, we analyse here the flow of glasses and glass–liquid transition in conditions of continuous electron irradiation such as under the e-beam of transmission electron microscopes (TEM) utilising the configuron (broken chemical bond) concept and configuron percolation theory (CPT) methods. It is shown that in such conditions, the fluidity of glasses always increases with a substantial decrease in activation energy of flow at low temperatures and that the main parameter that controls this behaviour is the dose rate of absorbed radiation in the glass. It is revealed that at high dose rates, the temperature of glass–liquid transition sharply drops, and the glass is fully fluidised. Numerical estimations show that the dose rates of TEM e-beams where the silicate glasses were fluidised are many orders of magnitude higher compared to the dose rates characteristic for currently vitrified HLW.

## 1. Introduction

Nuclear waste management generically ending with waste disposal is crucial to ensure the sustainable utilization of nuclear energy [1,2,3,4]. To be disposed, the nuclear waste is first transformed into a passive safe state which is typically achieved via immobilisation using durable waste forms (matrix materials with radionuclides confined in its structure). The waste form material is the primary barrier contributing to overall performance of storage, transportation, and final disposal [2,5,6]. Vitreous materials based on alkali–boron–silicate (ABS) and to a lesser extent on sodium–aluminium–phosphate (NAP) glasses are currently used on an industrial scale to immobilise the highest by hazard high-level waste (HLW) generated during the reprocessing of used nuclear fuel [5,6,7]. Table 1 provides approximate oxide compositions of both ABS glasses used worldwide and NAP glasses which were used at the Pamela plant in Belgium and are currently used in Russia to immobilise HLW.

**Figure 1:**
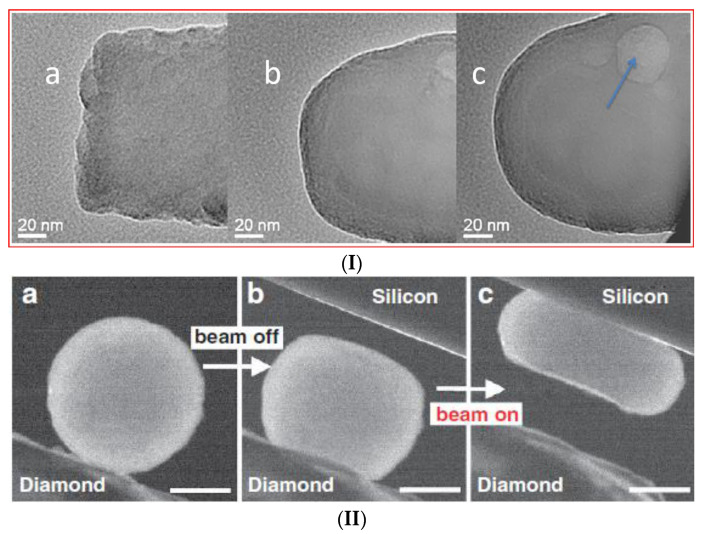

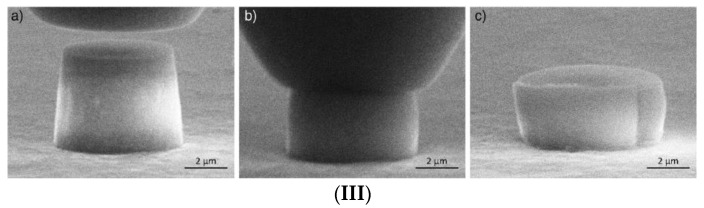
Reported examples of silicate glass fluidization irradiated by electrons. (**I**) Transformation of the rough end of a fractured Ca–alumina–borosilicate glass fibre (**a**) into a rounded fibre end (**b**) and, finally, a spherical glass bead (**c**) under electron irradiation in a moderately focused beam (~1000 nm diameter); JEM-2010F FEGTEM, total irradiation time 30 min. Arrows indicate the (transient) formation of bubble-type contrast (see reference [27] for details). Copyright Elsevier 2010; (**II**) Nano-compression of amorphous silica particle inside a transmission electron microscope (TEM). Two consecutive compression runs were performed with the electron beam being off and on, respectively. Panels (**a**–**c**) show the cantered dark-field images. In (**a**), the particle is adhered to the diamond punch and the silicon has not moved into the picture. In (**b**), the particle is imaged after it has been pressed with the beam off. Subsequently, with the beam on, the particle can be compressed into a pancake with a moderate force, as seen in (**c**). The scale bars are for 200 nm (see open access reference [25] for details). (**III**) Micro-pillar compression of silica glasses under a 20 kV and an 11 A/m^2^ electron beam at three stages: (**a**) before, (**b**) during and (**c**) after compression (see open access reference [26] for details).

The analysis of the effects of self-irradiation resulting from the decay of nuclear waste radionuclides is among the tasks of researchers investigating the long-term behaviour of vitrified HLW. Indeed, the role of self-irradiation in the behaviour of nuclear waste glasses is not fully understood with partly controversial results [8,22,23] and even unexpected radiation-induced effects reported on glasses irradiated externally by electrons [24,25,26]. Figure 1 demonstrates the radiation-induced fluidisation termed also quasi-melting with the drop of glass viscosity from that characteristic to solids to that one characteristic to the liquid state of matter as reported for tens of nano- [27], hundreds of nano- [25], and micro-metre [26]-sized glass samples.

Since the specimen temperatures on electron irradiation have remained practically unchanged (close to normal), the fluidisation of glasses is hence associated with a substantial drop of the glass transition temperature; moreover, this effect is independently reported by several research groups for silicate family glasses at sample sizes ranging from nano- to micrometres [24,25,26,27]. Fluidisation of highly radioactive glasses under irradiation is therefore an important topic which needs more attention along with other HLW immobilising materials [23]. Effects caused by irradiation of glasses by energetic electrons such as in electron microscopy is a topic of interest as an instrument to simulate the damage caused by the beta-decay of fission products on vitrified HLW [8,22]. A part of the electron beam energy is transferred to the specimen via electron–phonon scattering which causes specimen heating whereas another part generates point defects in the structure of material. The damage caused by irradiation can be classified as radiolysis and knock-on damage [28] where the radiolysis involves change in the electronic structure and includes the breaking of the chemical bonds and knock-on damage results in a direct displacement of atoms via kinetic energy transfer. In both cases, one or more displacements of atoms from their initial structural positions occur. The crucial question related to this aspect is whether the dose rates of vitrified HLW can suffice to cause effects like glass transition and viscosity drops to attain levels which may be of concern for radionuclide migration. Early assessments showed that in scanning electron microscopes (SEM), the dose rate was 2.7 × 10^3^ rad/s (27 Gy/s) for silica films irradiated by electrons at a 30 kV e-beam current of 100 pA, raster area of 0.1 cm^2^ and scan time of 1 s [29], which seems to be comparable with the values of vitrified HLW being initially of the order of 1.4 × 10^2^ Gy/s [30] although decreased after 30 years of storage to about 0.3 Gy/s [31]. As for transmission electron microscopy (TEM), it is noted that only a small fraction of electrons is absorbed in the sample analysed; otherwise, imaging would be not possible. Careful assessments show that on limited current densities of electron beams, the energy deposited in samples analysed both in SEM and TEM is typically very small and as a rule causes a negligible rise of sample temperatures [24,27].

The purpose of this work is to briefly analyse the flow and the glass–liquid transition in silicate glasses and compare the critical parameters of irradiation aiming to reveal the threshold levels when these effects might eventually occur in HLW glasses. This is achieved using the configuron percolation theory (CPT) [32]) which is based on the concept of broken chemical bonds termed amorphous material configurons [33,34].

## 2. Flow of Glasses

**Figure 2:**
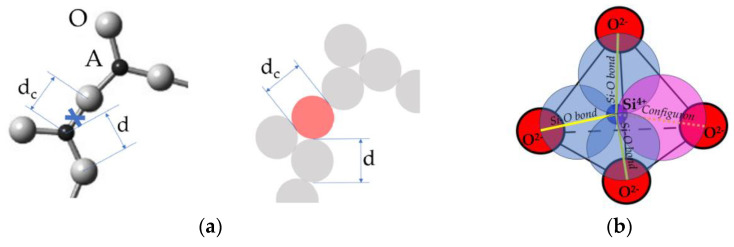
Schematic of a configuron in A_2_O_3_ and silicate glasses. (**a**) Configuron formation in the covalently bonded A_2_O_3_ glasses where A is a trivalent element such as Al or B (shown in light red). Its diameter d_c_ is not equal to the initial bond length d (see open access reference [35] for details). (**b**) Configuron formation on breaking a Si-O bond in silicate glasses.

Materials flow is caused by applied stress, designated F. The ability to flow is characterised by the viscosity coefficient η(T), which relates the acting stress to the strain rate ε ˙ linearly F = η(T)⋅ε ˙. The ease of flow termed fluidity is defined as the reciprocity of viscosity φ(T) = 1/η(T). It depends on material composition and external parameters such as pressure, temperature, intensity of radiation, e.g., laser beam or ionising photonic or particle irradiations. Generically, the fluidity of amorphous materials increases with the intensity of applied external parameters, e.g., the higher the temperature, the higher the fluidity. Such type of fluidizing behaviour is explained by the forced breaking of the chemical bonds between molecular building blocks of condensed matter which can be simple monoatomic (metals, noble gases) or polyatomic (oxides, chalcogenides, polymers, biological species, macroscopic blocks such as sand particles) entities. Here, we analyse the flow based on the system of chemical bonds which hold these blocks together by including in the analysis the system of broken chemical bonds termed configurons [20,21,22,23,24,25,26,27,28,29,30,31,32,33,34]. Indeed, following the Angell and Rao analysis of covalently bonded materials [33], we can define a unique chemical bond lattice which is congruent to the atomic lattice, and is either regular in the case of crystals or aperiodic in the case of quasicrystals, and disordered for amorphous materials independently of their nature. A configuron is defined as an elementary configurational excitation which involves breaking of a chemical bond and associated strain-releasing local adjustment of centres of atomic vibration [33,34]; see Figure 2.

The concept of configurons was generalised by Egami et al. [36] for metallic glasses and is being used to describe the glass transition phenomena in metallic systems [37,38,39]. The higher the temperature of an amorphous material, the higher the configuron concentration, and because the configurons weaken the bond system, the higher the content of configurons, the higher the fluidity. Indeed, as suggested by Mott [40], the flow is mediated by flow defects facilitating atomic motion, hence the fluidity is proportional to the concentration (*C*) of flow defects $\varphi \left(T\right)=\left(\gamma v{)}^{2}\nu C\;exp(-H/kT\right)/kT$, where *H* is the activation enthalpy of flow, *k* is the Boltzmann’s constant, *γ* is the shear strain produced by the motion of a single defect, *v* is the volume of the defect, and *ν* is an attempt frequency which has the order of the Debye frequency. Moreover, at very high concentrations, configurons can form percolation clusters: this means that the material loses its rigidity as it becomes penetrated by a macroscopic (theoretically infinite size) cluster made of broken bonds in line with the Kantor and Webman proof that the rigidity threshold of an elastic percolating network is identical to the percolation threshold [41]. The glass–liquid transition is thus considered as a percolation-type phase transition in the system of configurons [32].

The configurons weaken the disordered lattice of glass forming materials which results in generically higher fluidity at higher temperatures. This feature of condensed matter behaviour is opposite to temperature behaviour of gases where atoms or molecules are almost free, and their fluidity decreases with temperatures. That suggests that the viscosity of matter has a minimum value, a fact which was theoretically derived by Trachenko and Brazhkin based on fundamental considerations [42] and demonstrated within CPT for organics in [43].

We first consider the concentration of broken bonds *C*(*T*) in the absence of irradiation when its dependence on temperature is given in by the following equation [34]:

(1) $$C\left(T\right)=C_{0}\;exp\left(-G_{d}/RT\right)/\left[1+exp\left(-G_{d}/RT\right)\right],$$

where *G_d_* = *H_d_* − *TS_d_* is the Gibbs free energy configurons formation, *H_d_* and *S_d_* are the corresponding enthalpy and entropy of configurons formation, and *C*_0_ is the concentration of unbroken bonds at absolute zero (at which ideally no configurons exist in the unirradiated material). The viscosity is related to the diffusion coefficient *D* via the Stokes–Einstein equation:

(2) $$\eta \left(T\right)=kT/6\pi rD,$$

where *r* is the radius of configuron. The motion of configurons in the material occurs via jumps between different energy minima in the existing potential energy landscape associated with disordered chemical bond lattice sites where the configurons are in equilibrium positions. Gibbs free energy *G_m_* which is required to enable the configuron to jump across a barrier equals the difference in energy between the energy associated with the configurons being in equilibrium positions and the energy associated with the diffusing configuron along with its neighbours, which move apart to allow the jump, being in a saddle point configuration at a maximum in the energy–distance curve. The probability of the energy gathered through thermal fluctuations is given by the Gibbs distribution:

(3) $$w=exp\left(-G_{m}/RT\right)/\left[1+exp\left(-G_{m}/RT\right)\right],$$

where *G_m_ = H_m_ − TS_m_* is the Gibbs free energy of motion of configurons, and *H_m_* and *S_m_* are the corresponding enthalpy and entropy of configuron motion. Assuming that the mean jump time of configurons is short compared to the mean residence time of configurons *τ*(*T*) in their network sites, the trajectory of a configuron is composed of a sequence of elementary jumps with average jump length λ. From these microscopic quantities, the configuron diffusion coefficient can be defined by equality *D* = *fg*λ^2^*ν*(*T*), where *f* is the correlation factor, *g* is a geometrical factor close to 1/6 and *ν*(*T*) = 1/*τ*(*T*) is the total jump frequency. The correlation factor equals unity for purely random hopping although generically it is within the range 0 < *f <* 1. For defect mediated jumps, the equation for the total jump frequency is given by *ν*(*T*) = *zp*_0_*C*(*T*) *ν*_0_*w*/*C*_0_, where *z* is the number of nearest neighbours, *p*_0_ is a configuration factor which in simple cases is *p*_0_ = 1, and *ν*_0_ is the configuron vibrational frequency or the frequency with which the configuron attempts to surmount the energy barrier jumping into a neighbouring site. This frequency is of the order of the Debye frequency. Therefore, in the absence of irradiation, the viscosity of amorphous materials is given by the two-exponential equation [32,34,44]:

(4) $$\eta \left(T\right)=A_{1}T\left[1+A_{2}\;exp\left(B/RT\right)\right]\left[1+C\;exp\left(D/RT\right)\right],$$

where *A_1_* = *k*/6*πrD*_0_, *D*_0_ = *fg*λ^2^*zp*_0_*ν*_0_, *A*_2_ = *exp*(−*S_m_*/*R*), *B* = *H_m_*, *C* = *exp*(−*S_d_*/*R*), *D* = *H_d_*. Having five fitting parameters (*A*_1_, *A*_2_, *B*, *C*, *D*), Equation (4) describes well all the available experimental data on viscosities of amorphous materials without restriction of temperature intervals including both glassy and molten states of matter. It should be noted, however, that organic molecular liquids at higher temperatures exhibit a variation of *H_m_* from higher to lower values presumably due to thermal expansion [43]. In practice, Equation (4) shows numerically the same results as those of widely used models such as the VFT equation within the restricted temperature intervals. The experiments show that as a rule, *A*_2_*exp*(*B*/*RT*) >> 1; therefore, Equality (4) simplifies to a four-parameter equation

(5) $$\eta \left(T\right)=AT\;exp\left(B/RT\right)\left[1+C\;exp\left(D/RT\right)\right],$$

where *A* = *A*_1_*A*_2_ [44]. It is notable that in contrast to many approximations including VFT Equations (4) and (5) can be used over wider temperature ranges and provide correct Arrhenius asymptotes at high and low temperatures, namely $\eta \left(T\right)≅AT\;exp(B/RT)$ and $\eta \left(T\right)≅ACT\;exp[\left(B+D\right)/RT],$ respectively. The activation energy of the flow at low temperatures (in the glassy state) is high and given by *Q_H_ = B + D*, whereas at high temperature (in the melts) the activation energy of the flow is low and given by *Q_L_ = B*. Volf provides data for asymptotic activation energies of the viscous flow in glass forming liquids as follows: *Q_L_* = 80–300 kJ/mol for high temperatures (at low viscosities when *η* < 10^3^ dPa·s), and *Q_H_* = 400–800 kJ/mol for low temperatures in melts and glasses (at high viscosities when *η* > 10^3^ dPa·s) [45]. The fitting parameters of Equation (4) or its simplified version (5) are known for few glasses, e.g., for vitreous silica these are as follows: *A*_1_ = 1.15 × 10^−5^, *A*_2_ = 1.49 × 10^−6^, *B* = 522 kJ/mol, *C* = 2.42 × 10^−8^, *D* = 236 kJ/mol; see, e.g., refs [34,43,44]. These parameters were never identified for nuclear waste glass compositions (see Table 1), although methods to calculate them are developed and their estimation would require data on viscosity at two temperatures below and two temperatures above the glass transition temperature, *T_g_* [46,47].

## 3. Viscosity under Irradiation

The higher the configuron concentration, the higher the fluidity, i.e., the lower the viscosity. In addition to temperature fluctuations causing configuron generation at equilibrium concentration given by (1), other processes can also cause bond breakages and so the increase in fluidity, e.g., irradiation with particle (photons, electrons, protons, etc.) energies exceeding bond strength results in interatomic bond breaking and therefore diminish the viscosity. The higher the intensity of irradiation, the higher the rate of configuron generation; thus, the higher the fluidity. To quantify the increase in fluidity, let us first analyse the configuron concentration under irradiation and then follow the approach described above, aiming to derive an equation of viscosity in conditions of irradiation.

The radiation-induced defects such as broken chemical bonds generated by radiation in condensed matter are not thermalised and tend to annihilate returning to their equilibrium state. Before relaxing to the equilibrium state, they pass through several metastable states which takes some time; it should be noted that for solids such as glasses the relaxation can be rather slow. We consider here the situation of continuous irradiation with a certain constant dose rate *P_R_* = *const* when a stationary (although non-equilibrium) state is established with configuron concentration not depending on time. In this case, we can describe the viscous flow in a steady state approximation if the relaxations have significantly shorter characteristic times than the lifetime of radiation-induced configurons and that the fluidity adiabatically follows changes in the concentration of configurons contributing to viscous flow. In this case, the concentration of configurons in the irradiated material *C_i_* is the sum of configurons created by thermal activation given by (1) and of those created by radiation, *C_R_*. Due to structural changes, both formation and motion Gibbs free energies of configurons in an irradiated material (*G_di_* and *G_mi_*) can be different from those of a non-irradiated material (*G_d_* and *G_m_*), particularly at enhanced dose rates of radiation, *P_R_*. We can, however, assume as a first approximation that the irradiated material has the same structure except additional broken bonds, i.e., *G_di_* ≈ *G_d_* and *G_mi_* ≈ *G_m_*, and directly use Equation (1) for thermally generated configurons accounting for configuron parameters derived from the existing experimental data on viscosity [44]. In the simplest model with *C_R_* << *C*_0_ with only one effective recombination channel, the kinetics of radiation-induced configuron generation is described by equation

(6) $$\frac{dC_{R}}{dt}=k_{b}C_{0}P_{R}-\frac{C_{R}}{\tau_{b}},$$

where *k_b_* is the rate constant of bond breaking by radiation, *P_R_* is the intensity of radiation (absorbed dose rate, Gy/s), and *τ_b_* is the lifetime of a broken bond created by radiation. In the steady state approximation, *dC_R_/dt* = 0, and so the concentration of radiation-induced configurons is given by

(7) $$C_{R}=k_{b}C_{0}P_{R}\tau_{b}.$$

In this approximation, the configuron concentration is directly proportional to the intensity of radiation *P_R_*, although generically, radiation-induced defect concentrations depend on parameters of creation–recombination channels available and are often nonlinear with dose [48]. The viscosity of irradiated amorphous matter can thus be obtained accounting for the sum of configurons given by both (1) and (7), resulting in the following equation:

(8) $$\eta_{R}\left(T\right)=\eta \left(T\right)/\left[1+k_{b}P_{R}\tau_{b}\left[1+C\;exp\left(D/RT\right)\right]\right].$$

**Figure 3:**
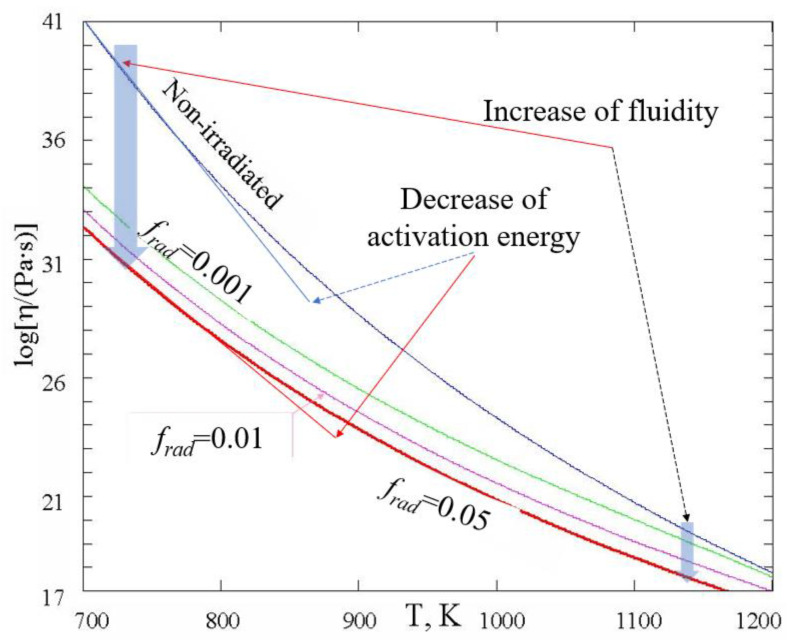
Temperature dependence of viscosities of non-irradiated and irradiated amorphous silica at three increasing dose rates. Here, the dimensionless dose rate is *f_rad_ = k_b_P_R_τ_b_*. Thermodynamic parameters of configurons were taken as those of non-irradiated silica glasses from [44].

Figure 3 shows the temperature dependence of viscosity at different intensities of radiation.

From (8) and Figure 3, one can see that the radiation is always increasing the fluidity. Since $\left[1+k_{b}P_{R}\tau_{b}\left[1+C\;exp\left(D/RT\right)\right]\right]>1,$ the viscosity of amorphous materials is always lower in conditions of irradiation. The higher the intensity of radiation *P_R_,* the higher the fluidity, although, contrary to the temperature dependence, the dose rate dependence is not asymptotically exponential. At very low levels of radiation, when *P_R_* → 0, the viscosity given by (8) reduces to that given by (4) or (5); however, at higher levels of radiation, the viscosity can be drastically decreased by radiation-induced configuron generation. The lower the temperature of glasses, the more significant the radiation-induced decrease in viscosity at the same level of radiation intensity *P_R_*, similarly to the effect of radiation on diffusion and leaching of alkalis in silicate glasses [49]. From (8), it follows that in practice, the decrease in viscosity is significant if the intensity of radiation is higher than the threshold level $P_{R}>P_{R}^{*}$ at a given temperature *T*, or, alternatively, the temperature is lower than the threshold temperature *T* < T*. The threshold dose rate and threshold temperature are given by formulae

(9) $$P_{R}^{*}=exp(S_{d}/R)\;exp(-H_{d}/RT)/k_{b}\tau_{b}\;\mathrm{and}\;T^{*}=H_{d}/\left[S_{d}-R\;ln(k_{b}P_{R}\tau_{b})\right].$$

The threshold dose rate increases exponentially with temperature with the lower $P_{R}^{*}$ at lower temperatures. The lower the rate constant of radiation-induced configuron generation and the lifetime of configuron created by radiation, the higher the intensity of radiation field required to observe the radiation-enhanced fluidity. Hence, the radiation-induced fluidity can be readily observed if the intensity of radiation is above $P_{R}^{*}$ and the temperatures are below T*. The higher the intensity of radiation *P_R_*, the rate constant of radiation-induced bond breaking (*k_b_*) and the lifetime of broken bonds created by radiation (*τ_b_*), the higher the critical temperature T*. At a temperature low enough so that *T* << T*, viscosity Equation (9) simplifies to equality

(10) $$\eta_{R}\left(T\right)≅\left(AT/k_{b}P_{R}\tau_{b}\right)\;exp\left(B/RT\right).$$

It can be seen from here that the activation energy of viscous flow in irradiated glasses becomes low *Q_L_* = *B* in this case. In the absence of radiation, such a type of flow is a characteristic of melts, i.e., above the glass transition temperature [32,34]. As the low activation energy of viscosity is equal to the enthalpy of motion of configurons, we can conclude that the radiation-enhanced flow is governed by configuron motion rather than by the bond breaking processes which conforms with experimental data [50] where the decrease in both viscosity and activation energy of flow were experimentally detected.

Finally, we emphasise that the nature of radiation is not specified in viscosity Equation (8), and hence the conclusions drawn are generic for all types of bond-breaking processes such as UV-laser or ionising radiations.

## 4. Glass Transition on Irradiation

The nature of glass transition is dual showing both kinetic and thermodynamic features [32,51,52]. In many instances, the transformation of a liquid into a glass (vitrification) is regarded as a transition for practical purposes rather than a thermodynamic phase transition. Thus, in the kinetic approach by general agreement, it is considered that on cooling, the liquid transforms into a glass when the viscosity is equal to 10^12^ Pa·s (10^13^ poise) or when the relaxation time is 10^2^ s [53]. Then, the glass transition temperature *T*_*g*,*relax*_ is determined from the viscosity–temperature relationship $\eta \left(T_{g,relax}\right)=10^{12}\left(\mathrm{Pa}\cdot s\right)$. As we have seen, the irradiation decreases the viscosity; thus, according to (8), the glass transition temperature of irradiated materials decreases with the increase in irradiation dose rate *P_R_*. In the thermodynamic approach, the glass–liquid transition is analysed based on the Kantor and Webman theorem which indicates that the lattice loses its rigidity at the percolation threshold of the bonding system [41]. Thus, within the CPT approach, the glass transition is considered as a percolation in the system of configurons [32,34,54]. The temperature of the glass–liquid transition is found via equalising the concentration of configurons to the threshold percolation concentration *f_c_* [54]: *C*(*T*_*g*_) = *f*_c_*C*_0_ from which the following expression is obtained:

(11) $$T_{g}=H_{d}/\left[S_{d}+R\;ln[\left(1-f_{c}\right)/f_{c}]\right].$$

The glass transition temperature *T_g_* of an irradiated material can be found assuming that the configuron concentration which is the sum of thermal and radiation-induced configurons achieves the universal critical density given by the percolation theory *C*_*i*_(*T*_*g*_) = *f*_*c*_*C*_0_. This leads to a reduced glass transition temperature under irradiation compared to that of unirradiated materials:

(12) $$T_{gi}=H_{di}/\left[S_{di}+R\;ln[\left(1-f_{c}+k_{b}P_{R}\tau_{b}\right)/\left(f_{c}-k_{b}P_{R}\tau_{b}\right)]\right].$$

The higher the radiation dose rate, the lower the glass transition temperature. Equation (12) demonstrates that that at irradiation dose rates higher than the threshold level given by equality

**Figure 4:**
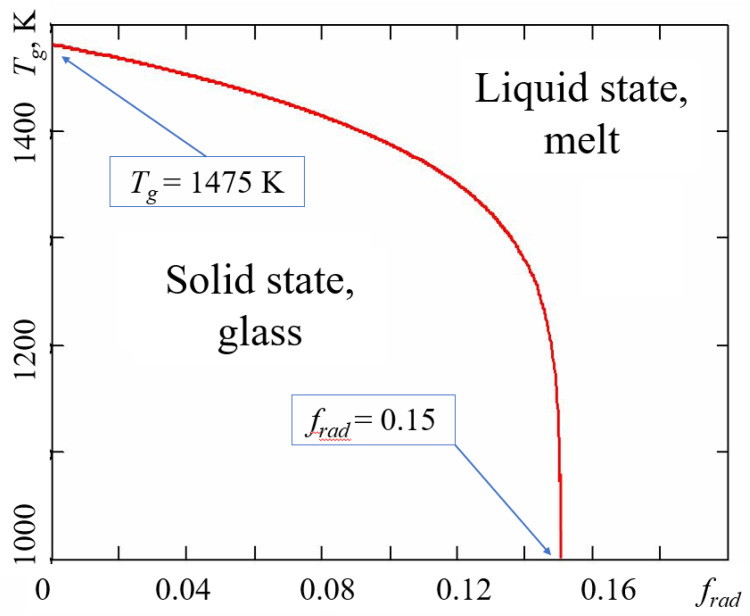
Glass transition temperature of irradiated amorphous silica as a function of dimensionless radiation dose rate *f_rad_ = k_b_P_R_τ_b_*. Thermodynamic parameters of configurons were taken as those of non-irradiated silica glass from [44].

*P_c_* = *f_c_*/*k_b_τ_b_*, (13)

the glass transition temperature drops to zero, meaning that the irradiated material is fully melted, even when kept close to zero degrees Kelvin. Naturally, the glassy material transforms to liquid because of intensive radiation-induced bond breaking, whereas thermal fluctuations do not generate any configurons. Figure 4 shows the results of glass transition temperature calculations for irradiated amorphous silica.

It is seen from Figure 4 that indeed if the intensity of radiation is higher than the threshold level *P_c_* = *f_c_*/*k_b_τ_b_*, where in the case of silica *f_c_* = 0.15, the vitreous material is completely melted by radiation.

Fragile glassy materials have lower percolation thresholds *f_c_* << 1 [43,51]; hence, these materials can be melted at significantly lower intensities of radiation. Note that fluidised glass melts are still quite viscous so that in order to experimentally detect the viscous flow, very small, e.g., sub-micrometre or nano-scale size samples are required such as those studied by Ajayan and Iijima [55], Moebus et al. [27], Zheng et al. [25], and Bruns et al. [26].

## 5. Irradiation Dose Rates

The crucial question related to nuclear waste glasses is whether the dose rates within the masses of vitrified HLW can be as high as those in the experiments with glass fluidization reported so far or not. The self-irradiation of HLW glasses occurs due to the decay of waste radionuclides and this is causing a significant increase in temperature, imposing the need for waste canisters cooling in the initial storage period lasting for several decades. During the first several hundred years after waste vitrification, radiation emissions in nuclear waste glasses are dominated by beta and gamma radiation arising from the decay of fission products such as ^137,134^Cs, ^90^Sr [8,22]. Table 2 shows the radiation types within vitrified HLW with the resulting doses of absorbed energy from them and displacements of atoms from their initial positions in the material [8,22]; moreover, it is worth noting that the displacement of atoms is causing their effective delocalisation and finally can result in the transformation of glass into liquid, i.e., its effective melting caused by delocalisation of atoms [52].

The high dose rates gradually decrease with time, e.g., the dose rate at the contact of vitrified HLW canister decreases from 872 Gy/h after 30 years from vitrification to 0.07 Gy/h after 500 years [31]. The dose rates are significantly higher immediately after HLW vitrification, e.g., the IAEA technical report offers surface dose rates of approximately 5·10^5^ R/h from a 60 L canister containing HLW vitrified waste at the Pamela plant (Belgium) containing up to approximately 200 kCi of ^137^Cs and 150 kCi of ^90^Sr, with 2.0 kW of decay heat [30]. Hence, currently, vitrified HLW can be characterised by initial dose rates *P_R_* of the order of 1.4 × 10^2^ Gy/s [30] or after 30 years of storage 0.3 Gy/s [31]. Data from Table 2 reveal that for the initial period of time, which lasts about 10^4^ years, the absorbed dose of radiation is almost entirely due to the absorption of beta particles which emphasises the dominant role of electron radiation in this period of vitrified HLW.

The dose rate of irradiates glass samples in the SEM/TEM *e*-beam can be found via calculating the energy loss of beam Δ*E* in the irradiated glass sample which we consider here as a disc of radius *r_s_* having thickness *d_s_*. Its corresponding mass is then *m_s_* = *π*(*r_s_*)^2^*d_s_ρ_s_*, where *ρ_s_* is the density of glass. The absorbed dose rate of radiation by definition is the energy loss per unit of time per mass *P_R_* = Δ*E*/*m*_*s*_*t*. The energy loss is negligible compared to the initial energy of electrons Δ*E* << *E*. It can be calculated using the following equation:

(14) $$∆E=N_{e}\int_{0}^{d_{s}}\frac{dE}{dx}dx,$$

where *N_e_* is the number of electrons passing through the sample and *dE/dx* is the stopping power for the electrons in the sample. The stopping power of the electrons is typically calculated using the Bethe–Bloch equation [24,27,56,57,58]. The average stopping power in silicates was assessed as 〈*dE*/*dx*〉 ≈ 0.5 ÷ 1.0 eV/nm [29,56,57,58]; hence, we can approximately estimate the dose rate of TEM *e*-beam irradiated samples as

(15) $$P_{R}\approx \frac{i_{s}dE/dx}{\rho_{s}e},$$

where *i_s_* = *N_e_e*/*π*(*r*_*s*_)^2^*t* is the density of the current passing through the sample, *e* is the electron charge. From (14), we can determine that for silica glasses (*ρ_s_* = 2.2 g/cm^3^), the absorbed dose rate of electron radiation in silicate glass samples is about (2–4) × 10^3^ Gy/s at a typical electron microscopy density current in the sample *i_s_* = 1 nA/cm^2^.

## 6. Discussion

Utilising the CPT approach, we obtained explicit equations of glass viscosity and glass transition temperature in the conditions of continuous electron irradiation, which demonstrates that both viscosity and glass transition can be significantly affected by radiation with the main controlling parameter being dose rate *P_R_*. The higher the *P_R_*, the lower the viscosity (Equation (8) and Figure 3) and the lower the glass transition temperature (Equation (12) and Figure 4).

Electron irradiation is also causing a decrease in the activation energy of the viscous flow in glasses (Equation (10) and Figure 3). It is notable that these generic conclusions drawn within CPT conform well to the experimental data available. Apart from the explicitly visualised effects of the flow induced by TEM electron irradiation (Figure 1), there have been many observations of radiation-induced effects in glasses such as variations in refractive index, density and mechanical properties [8,22], e.g., fused silica undergoes extensive densification on prolonged exposure to high-energy neutron, electron, and γ-ray radiation [59]. The densification of fused silica occurs also upon exposure to laser radiation [60]. Both high-energy radiation and laser-radiation-induced densification involve the weakening of interatomic bonds and subsequent relaxation effects so that the densification follows a universal function of the dose. Silica glass densification is caused by radiation-induced breaking of bonds and subsequent rearrangements of the SiO_2_ ring network into compacter rings with the density eventually saturated with fluence [59]. Ion irradiation of amorphous solids revealed stress relaxation and surface smoothing, and also demonstrated viscous flow below melting temperatures [50,61].

The viscosity of ion-beam irradiated amorphous silicon was estimated as 10^13^ Pa·s, which is about 10^4^ smaller compared with the viscosity of non-irradiated material. This was explained by the creation of broken bonds by radiation [50]. It was found that the radiation-enhanced fluidity increases with increasing radiation energy loss (or ion mass) and is approximately proportional to the energy loss [61], which looks in line with the conclusions drawn from Equation (9) and Figure 3. Moreover, it was found that the activation enthalpy of the flow of irradiated silicon was smaller than 0.3 eV; in comparison, that of non-irradiated material is 1.8 eV (this is roughly the energy required to break a bond). This difference in activation energy demonstrated that the radiation-enhanced flow is not governed by bond breaking but rather by bond motion [50]. These results thus conform well to the conclusion from Equation (10). Studies of electron-beam-induced sintering of sub-micrometre silicon particles demonstrated that the viscosity of amorphous silica drastically decreased by many orders of magnitude in a 200 kV TEM electron beam of a 10 A/cm^2^ current density [55]. Although the temperature was estimated in those experiments as not higher than a few hundred degrees, the viscosity was as low as 10^8^–10^9^ Pa s, which would otherwise need temperatures above 1700 K. Such low viscosity of irradiated amorphous silica was attributed in [55] to the increase in defect concentration associated with the local structure. Molecular dynamics simulations have also demonstrated that point defects (Frenkel pairs) provide an efficient mechanism for radiation-induced viscous flow of solids [62]. By simulation of the injection of interstitial and vacancy-like defects, it has been demonstrated that point defects induce the same amount of flow as the recoil events, indicating that point-defect-like entities mediate the flow process in solids even at 10 K. It was concluded that the radiation-induced flow does not require thermal spikes (local melting) and that point defects equally, or, in many cases, more efficiently provide the viscous flow, which earlier has been associated with thermal spikes (local melting) [62].

CPT approach to viscosity and glass transition revealed that significant changes in fluidity compared with non-irradiated samples occur either when the dose rate exceeds the certain threshold level or if the temperature of the sample is low enough (Equation (9)). This is physically explained by the fact that in these conditions (*P_R_* > P_R_* or *T* < T*), the concentration of configurons generated by radiation becomes higher compared to the concentration of thermally activated configurons. The transformation of glass into liquid in conditions of electron irradiation is characterised by a very sharp drop of glass transition temperature when the dose rate *P_R_* attains the critical value *P_c_* given by Equation (13). Within CPT, we thus draw the conclusion that the crucial parameter of electron irradiation is the dose rate. Table 3 shows the estimated dose rates of absorbed radiation of TEM *e*-beam irradiated glass samples which demonstrated fluidization.

We can now compare the results obtained considering the case of vitrified HLW. As can be seen, the dose rates of sample silicate glasses irradiated by *e*-beams are many orders of magnitude (4–7 orders) higher than the dose rates of currently vitrified HLW which have the order of several hundred Gy/s. This implies that the fluidisation is not the case and not possible within vitreous wasteforms used industrially; however, this indicates that significant increases in nuclear waste loadings of HLW glasses have limits where dangerous processes may start occurring.

Apart from the case of HLW, fluidisation of amorphous materials in radiation fields could be effectively used for some technological purposes such as nanopatterning, welding, sintering and surface smoothing of materials [63,64,65] as well as for high-intensity dosimeters required for new generation of nuclear reactors and radiation devices [66]. It is notable that the radiation used for non-thermal fluidisation can be high-energetic (ionising), such as in the case of the electron radiation analysed here, of the proton, ion, neutron, γ-ray type, or photonic, such as that generated by a laser.

## 7. Conclusions

Intensive electron irradiation of glasses causes the increase in their fluidity and a decrease in glass transition temperature. Explicit equations of material viscosity and glass transition temperature can be readily derived using the CPT modelling of amorphous materials which analyses their system of chemical bonds (Equations (10) and (11)). The key parameter controlling the increase in fluidity and the decrease in glass transition temperature is shown to be the absorbed dose rate of radiation. Sensible decrease in viscosity can be revealed either when the dose rate exceeds a certain threshold level or when the temperature is low enough (Equation (9)) so that the concentration of configurons generated by radiation exceeds it due to thermal excitations. Numerical estimations of dose rates in experiments where the fluidisation of glasses was experimentally observed show that they exceed the dose rates of radiation characteristic for vitrified HLW by many orders of magnitude. The flow and glass transition induced by electron irradiation can be useful in various applications where technological operations at nano- and micro-size scale are needed such as nano- and micro-welding, surface polishing and smoothening.

## Figures and Tables

**Table 1:** Oxide compositions of HLW glasses (wt.%) [7,8,9,10,11,12,13,14,15,16,17,18,19,20,21].

Country	Facility	Composition
Belgium	Pamela	70.7P_2_O_5_·7.1Al_2_O_3_·22.2Fe_2_O_3_ 52.7SiO_2_·13.2B_2_O_3_·2.7Al_2_O_3_·4.6CaO·2.2MgO·5.9Na_2_O·18.7 Misc. ^1^
France	AVM Marcoule	46.6SiO_2_·14.2B_2_O_3_·5.0Al_2_O_3_·2.9Fe_2_O_3_·4.1CaO·10.0Na_2_O·17.2 Misc.
France	AVH R7/T7 La Hague	54.9SiO_2_·16.9B_2_O_3_·5.9Al_2_O_3_·4.9CaO·11.9Na_2_O·5.5 Misc.
Germany	Karlsruhe	60.0SiO_2_·17.6B_2_O_3_·3.1Al_2_O_3_·5.3CaO·7.1Na_2_O·6.9 Misc.
Japan	Tokai Vitrification Facility	46.7SiO_2_·14.3B_2_O_3_·5.0Al_2_O_3_·3.0CaO·9.6Na_2_O·21.4 Misc.
India	WIP Trombay	30.0SiO_2_·20.0B_2_O_3_·25.0PbO·5.0Na_2_O·20.0 Misc.
India	AVS Tarapur	34.1SiO_2_·6.4B_2_O_3_·6.2TiO_2_·0.2Na_2_O·9.3MnO·43.8 Misc.
Russia	EP500 PA “Mayak”	53.3P_2_O_5_·15.8Al_2_O_3_·1.6Fe_2_O_3_·23.5Na_2_O·5.8 Misc.
UK	WVP Sellafield	47.2SiO_2_·16.9B_2_O_3_·4.8Al_2_O_3_·5.3MgO·8.4Na_2_O·17.4 Misc.
US	DWPF Savannah River	49.8SiO_2_·8.0B_2_O_3_·4.0Al_2_O_3_·1.0CaO·1.4MgO·8.7Na_2_O·27.1 Misc.
US	WVDP West Valley	45.8SiO_2_·8.4B_2_O_3_·6.1Al_2_O_3_·11.4Fe_2_O_3_·1.4MgO·9.1Na_2_O·17.8 Misc.
US	WTP Hanford (under construction)	50.0SiO_2_·20.0B_2_O_3_·5.0Al_2_O_3_·25.0Na_2_O

^1^ Miscellaneous, includes waste oxides.

**Table 2:** Types of radiation and their resulting displacement effects in HLW glasses [8,22].

Type of Radiation	Range of Defects in Glass	Dose after 10^4^ Years, Gy	Dose after 10^6^ Years, Gy	Atomic Displacements per Decay
4–6 MeV α-particles	∼20 μm	∼3·10^9^	∼10^10^	∼200
∼0.1 MeV recoil nuclei	∼30 nm	∼6·10^7^	∼3·10^9^	∼2000
β-particles	∼1 mm	∼3·10^9^	∼4·10^9^	1
γ-irradiation	∼2 cm	∼2·10^9^	∼2·10^9^	<<1
(n, α) nuclear reactions	∼1 m	∼2·10^2^	∼3·10^3^	∼200–2000

**Table 3:** Estimated dose rates of *e*-beam irradiated glasses.

*e*-Beam Current Density *i_s_*, A/m^2^	Material	Dose Rate *P_R_*, Gy/s	Reference
10^4^	Calcium–alumina–borosilicate glass	(2–4) × 10^9^	[24]
180	Silica glass	(1–2) × 10^7^	[25]
11	Silica glass	(2–4) × 10^6^	[26]

## Data Availability

Data supporting reported results can be found within manuscript and references provided.
